# The potential risk of using historic claims to set bundled payment prices: the case of physical therapy after lower extremity joint replacement

**DOI:** 10.1186/s12913-022-08410-7

**Published:** 2022-08-19

**Authors:** Sander Steenhuis, Geeske Hofstra, France Portrait, Fatima Amankour, Xander Koolman, Eric van der Hijden

**Affiliations:** 1 Department of Health Sciences, Health Economics Section, Talma Institute, VU University Amsterdam, De Boelelaan 1085, 1081 HV Amsterdam, The Netherlands; 2 Department of Health Sciences, Health Economics section, VU University Amsterdam, De Boelelaan 1085, 1081 HV Amsterdam, The Netherlands; 3 Care Procurement Department, Zilveren Kruis, Storkstraat 12, 3833 LB Leusden, The Netherlands

**Keywords:** Bundled payments, Claims data, Pricing methods, Care procurement, Supplemental insurance, Total joint arthroplasty, Andersen model

## Abstract

**Background:**

One of the most significant challenges of implementing a multi-provider bundled payment contract is to determine an appropriate, casemix-adjusted total bundle price. The most frequently used approach is to leverage historic care utilization based on claims data. However, those claims data may not accurately reflect appropriate care (e.g. due to supplier induced demand and moral hazard effects). This study aims to examine variation in claims-based costs of post-discharge primary care physical therapy (PT) utilization after total knee and hip arthroplasties (TKA/THA) for osteoarthritis patients.

**Methods:**

This retrospective cohort study used multilevel linear regression analyses to predict the factors that explain the variation in the utilization of post-discharge PT after TKA or THA for osteoarthritis patients, based on the historic (2015–2018) claims data of a large Dutch health insurer. The factors were structured as predisposing, enabling or need factors according to the behavioral model of Andersen.

**Results:**

The 15,309 TKA and 14,325 THA patients included in this study received an average of 20.7 (SD 11.3) and 16.7 (SD 10.1) post-discharge PT sessions, respectively. Results showed that the enabling factor ‘presence of supplementary insurance’ was the strongest predictor for post-discharge PT utilization in both groups (TKA: β = 7.46, SE = 0.498, *p*-value< 0.001; THA: β = 5.72, SE = 0.515, *p*-value< 0.001). There were also some statistically significant predisposing and need factors, but their effects were smaller.

**Conclusions:**

This study shows that if enabling factors (such as supplementary insurance coverage or co-payments) are not taken into account in risk-adjustment of the bundle price, they may cause historic claims-based pricing methods to over- or underestimate appropriate post-discharge primary care PT use, which would result in a bundle price that is either too high or too low. Not adjusting bundle prices for all relevant casemix factors is a risk because it can hamper the successful implementation of bundled payment contracts and the desired changes in care delivery it aims to support.

**Supplementary Information:**

The online version contains supplementary material available at 10.1186/s12913-022-08410-7.

## Background

Bundled payments are fundamentally different from traditional fee-for-service based payment models, in which providers are paid separately for each service they deliver. Instead, under bundled payment models providers assume accountability for the quality and costs of care over a set period of time and (ideally) across multiple settings [1, 2]. Research suggests that this can increase the coordination of care between providers, and can reduce fragmentation in the health care system, while the quality of care remains the same, or even increases. This could in turn lead to better health and experiences for patients, lower costs for payers and higher margins for providers [3–5].

Although a bundled payment contract with just one provider (e.g. by bundling all hospital Diagnosis Related Groups [DRGs] related to one specific health condition) can help to reduce implementation complexity in the beginning, the real potential of these contracts lays in bundling all providers involved in the full care cycle of a patient’s condition [6, 7]. One of the most significant challenges in implementing such a multi-provider contract, is to determine an appropriate, casemix adjusted, payment amount (i.e. the price) for the total bundle [8–10].

Literature shows that there are multiple approaches to determine the price of the total bundle. For example, the payment amount could be based on: cost benchmarks with other providers, care pathways, guideline based standards, a method called ‘time-driven activity based costing,’ or claims-based historic service use [11, 12]. Among these approaches, the latter seems to be the most frequently used in bundled payment models [13]. For example, the Bundled Payments for Care Improvement Initiative, the largest bundled payment program in the world, creates target prices for an episode of care based on the discounted sum of historic claims for services that were previously provided within the episode (i.e. a 2–3% discount, after updating for national spending trends) [14].

Using average historical costs to calculate the bundle price has several key advantages, such as operational ease and the potential for payers to address a broad range of episode types due to a large amount of existing data. However, historical costs may not reflect the ideal patient care for a given episode and therefore lack clinical face validity with providers [15, 16]. Additionally, although analyzing claims retrospectively is important for payers and providers to acquire an indication of the costs to deliver care, they may not accurately reflect appropriate care. On the one hand, the historic claims could represent a substantial amount of potentially avoidable utilization of care. For instance, this could be due to potential supplier-induced moral hazard (e.g. when providers induce more care services for patients with insurance) or a patient-induced moral hazard effect (e.g. when patients utilize more care because it is covered by their health insurance) [17, 18]. This would result in an overestimation of the appropriate price. On the other hand, those claims could lack certain high value services that have historically not been utilized by providers because they were not reimbursed for it (e.g. preventive care or e-health). This would in turn result in an underestimation of the appropriate price [9, 13]. Multiple studies have tested the assumption that historic claims data may inaccurately reflect appropriate care delivery, but, to our knowledge, none of those studies applied quantitative analyses to actually strengthen that assumption with statistical evidence [9, 13, 15, 16].

This study explores the assumption that average historical claims data may not accurately reflect necessary and effective care, by investigating this in the context of post-discharge physical therapy (PT) use after total knee arthroplasties (TKA) and total hip arthroplasties (THA) of osteoarthritis patients. Osteoarthritis is a progressive joint disorder in which patients experience lower quality of life as a result of pain and decreased mobility in primarily their knees and hips [19]. When nonsurgical treatments like medication and exercise therapy are no longer sufficient, a TKA or THA procedure - in which the damaged cartilage and bone surface is replaced by artificial implants – is considered a cost-effective intervention [20]. After the surgery, patients usually follow a rehabilitation program in a PT clinic that aims at preventing unfavorable outcomes and at further restoring the joint’s functionality [21]. Therefore, PT in this post-discharge setting can be an effective service for patients and is often included in TKA or THA care episodes [22, 23]. Under bundled payments these types of elective care episodes increasingly show decreased spending [5]. This is often due to a reduction in the use of post-acute care services like post-discharge PT [24, 25], which accounts for a substantial part of the total costs of TKA and THA episodes [26, 27] and demonstrates large variations across providers [14, 28–30]. There is also little scientific evidence on the factors associated with variations in post-discharge TKA- and THA-related PT sessions [31]. All this further complicates the use of average historical costs of care by payers to determine the total bundle price.

More specifically, this study examines the predictors of utilizing post-discharge primary care PT after a TKA or THA in patients with osteoarthritis, based on the historic claims data of a representative Dutch health insurance company. This is relevant for setting bundled payment prices because much of the variation in care utilization is only warranted if it can be explained by differences in relevant casemix factors (e.g. age and health status) [32]. If much of the variation is explained by other factors, such as insurance status, then average historic claims may not accurately reflect appropriate care delivery, which complicates the determination of an appropriate bundle price. This study provides valuable insights to payers, providers and other stakeholders involved in bundled payment implementation, by raising awareness that historic claims data may not accurately reflect appropriate care delivery.

## Methods

### Study sample

This retrospective cohort study used pseudonymized historic claims data provided by Zilveren Kruis, the largest health insurance company in The Netherlands. Previous studies have shown that, with a 30% market share, the general characteristics of patients (such as their age and sex) in the claims data of Zilveren Kruis are representative for the total Dutch population [33, 34]. All osteoarthritis patients who had undergone a TKA or THA between 2015 and 2018 were included and data was collected in the period ranging from 1 year prior to 1 year after the surgery. Additional data on patient, hospital and primary care PT clinic characteristics was retrieved from two national claims databases (Vektis and Statistics Netherlands/CBS). Patients were excluded if: (i) they were below the age of 18 at the time of surgery (as their PT costs were always covered through the compulsory basic health insurance), (ii) their claims data was incomplete because they switched to another insurer in the year prior to or after surgery, (iii) they passed away within a year after surgery, (iv) they had received more than one TKA or THA during the study period (as PT use could no longer be attributed to one specific surgery), or (v) there were no claims of any post-discharge primary care PT sessions in our dataset (e.g. because the patient received post-discharge PT in another (long-term) care setting, in which case the information on PT use is not recorded in our dataset). This study did not require permission from the Medical Ethics Review Committee because patient identification numbers were pseudonymized.

### Utilization of post-discharge PT

In both patient groups (TKA and THA) the number of post-discharge primary care PT sessions was examined as dependent variable. A session was defined as all PT sessions (in the clinic or at home) for which the physical therapist submitted a claim to the insurer under the diagnosis code for post-discharge TKA or THA treatment up to 1 year after the surgery. Post-discharge TKA and THA PT utilization was examined in two separate prediction models because TKA patients generally require more post-discharge PT sessions than THA patients to achieve optimal outcomes [35].

### Factors associated with post-discharge PT utilization

To examine the factors associated with post-discharge primary care PT utilization the Andersen Behavioral Model of Health Services Utilization was used [36–38]. This widely used theoretical model assumes that not only the factors that are related to a patient’s need for care (e.g. health and functional status), but also those that predispose (e.g. demographics) or enable (e.g. health insurance coverage) the patient to obtain such care, will influence patterns of health care utilization. Therefore this model was considered suitable for our research objective and to structure the study’s results.

#### Predisposing factors

Predisposing factors refer to a patient’s existing characteristics that could influence the utilization of health care services [37, 39]. Within the data of this study three predisposing factors were considered relevant to post-discharge PT utilization and included in the analyses: 1. Sex, because women with osteoarthritis tend to have a higher utilization of osteoarthritis-related care than men [40, 41], 2. Age, because older TKA and THA patients tend to have a longer rehabilitation period [42, 43], and 3. Socioeconomic status (SES), because if the costs of care usage is reimbursed through an insurance plan (as is the case for most post-discharge TKA or THA PT care in the Netherlands), then patients with a lower SES tend to utilize more care [44, 45]. This SES information was retrieved by merging the study sample using the four-digit postal codes of patients with the national database of Statistics Netherlands (CBS) and categorized in five groups, ranging from low to high SES.

#### Enabling factors

Enabling factors refer to external conditions which could facilitate a patient’s utilization of care services (e.g. insurance benefit design, travel time and provider quality) [37, 39]. Within the data of this study, eight enabling factors were considered relevant to post-discharge PT utilization and included in the analyses.

The following four patient-level variables were included: 1. The presence of supplemental insurance (SI) for PT care (operationalized as yes/no) because in the Netherlands most PT services are not covered by the basic health insurance, and a higher degree of supplementary (private) coverage is associated with more care utilization [46, 47], 2. Travel time to the hospital where the patient underwent the TKA or THA, and 3. Travel time to the patient’s PT clinic, because longer travel times are generally associated with less care utilization [48, 49]. 4: The number of non-TJA (total joint arthroplasty) related PT sessions between 6-12 months prior to surgery in order to assess the patient’s tendency to use PT in the recent past, as this has been shown to be associated with an increased use of PT in the future [39, 50].

The following two hospital-level variables were included: 1. Type of hospital, operationalized as a categorical variable with the five Dutch hospital types (i.e. general, specialized, academic and top clinical hospitals, and independent treatment centers). This classification is relevant because while all hospital types are able to perform a TKA or THA, academic and top clinical hospitals generally treat patients with more complex care needs than general hospitals and independent treatment centers [51]. 2: The TKA and THA procedure volume per hospital, which is relevant because a smaller volume of procedures has been associated with more short-term complications and a higher revision risk [52, 53].

Finally, the following two PT clinic-level variables were included: 1. The total volume of PT sessions per clinic per year as an indication of the size of the clinic, and 2. The contract type between the PT clinic and the insurer. During the study period the procurement policy of the insurer consisted of three separate preferred-provider pay-for-performance contracts (i.e. ‘standard,’ ‘basic,’ and ‘plus’). Compared to a ‘standard’ contract a ‘plus’ contract required higher quality standards (e.g. mandatory outcome measurement and average treatment volume benchmarks with peers) but also provided a 16% higher reimbursement fee per PT session. The assumption is that clinics with a ‘plus’ contract hold a higher quality standard and work more efficient (e.g. that PT utilization after TKA and THA would be lower) [54].

#### Need factors

Need factors refer to individual conditions that influence the likelihood that a patient will utilize health care services (e.g. pathology and comorbidities) [37, 39]. Within the data of this study five need factors were considered relevant to post-discharge PT utilization and included in the analyses: 1. The number of TJA-related pre-operative PT sessions in the 6 months prior to the surgery, which was expected to be inversely related to post-discharge PT utilization because pre-operative PT aims to improve functional status and familiarizes patients with joint exercises in order to maximize post-discharge outcomes [55, 56], 2: The average number of pre-operative home care hours per month in the 12 months prior to the surgery, under the assumption that more home care hours could indicate a less self-sufficient patient who may require more PT sessions during the rehabilitation process, 3: Diabetes (yes/no), 4: Cardiovascular disease (yes/no), and 5: Chronic obstructive pulmonary disease (COPD) (yes/no). These three prevalent comorbidities in TKA and THA patients were included based on Pharmaceutical Cost Groups (PCGs) in the data [57, 58], which is a Dutch medication use classification system based on the Anatomical Therapeutic Chemical Classification System [59].

### Statistical analyses

Means and frequency distributions were first reported per study group. Additionally, multicollinearity was examined using variance inflation factor (VIF) statistics. Multicollinearity was assumed at a VIF > 10 and if that was the case then one of the intercorrelated variables was excluded [60]. Then multilevel linear regression analyses were used to investigate potential predictors for post-discharge PT use after TKA or THA. Traditional regression analyses were assumed less suitable due to the potential nesting of patients within hospitals and PT clinics, which may result in dependency between observations [61]. The extent of nesting was deemed insignificant if clusters contained less than 10 patients. Likelihood ratio tests were computed to assess whether the model improved after correcting for nesting and then an intraclass correlation coefficient (ICC) was calculated to estimate what proportion of variance in post-discharge PT use could be explained by nesting.

Relevant predictors for post-discharge PT use were explored in two different models: model A solely included predisposing and need factors and in model B enabling factors were added. Model A was estimated separately because traditional risk adjustment methods generally only correct for differences in predisposing and need factors, not for enabling factors. The final predictors were selected using a backward selection procedure with an alpha of 0.05 and then the ICC was calculated. All statistical analyses were carried out using SAS 7.1.

### Sensitivity analyses

Within the time frame of the study the insurer’s SI reimbursement policy changed. From 2015 until 2017 there were 4 different SI plans with different coverage for TJA-related post-discharge PT sessions, ranging from 9 to 40 sessions per year [62–64]. In 2018 there were only 3 different SI plans, which all covered 20 PT sessions per year for TJA-related post-discharge PT (see the discussion section for more detailed information on Dutch reimbursement schemes for PT care during our observation period) [65]. To test the robustness of our results and the effect of this change in coverage, separate multilevel regression analyses were conducted for patients that underwent surgery in 2018.

## Results

### Descriptive statistics

After applying the exclusion criteria, our study sample consisted of 15,309 TKA patients and 14,325 THA patients (Table 1). On average, TKA patients received 4 more post-discharge primary care PT sessions (20.7) compared to THA patients (16.7). Beside the amount of received home care prior to surgery (9.3 hours for TKA and 6.5 hours for THA), all predisposing, enabling and need factors were similarly distributed between the two study groups. In both groups about half of the patients received TJA-related PT in the 6 months prior to surgery, and cardiovascular disease was the most common comorbidity. VIF statistics were found to be acceptable (VIF < 10) in all cases and, because of that, no variables were excluded due to multicollinearity.

**Table 1:** Descriptive characteristics of patients who received physical therapy after knee or hip replacement surgery (2015–2018)

Characteristic	TKA ***(n = 15,309)***	THA ***(n = 14,325)***
Mean number of post-discharge PT sessions (SD)	20.70 (11.3)	16.68 (10.1)
**Predisposing factors**		
Female (%)	63	66
Age (SD)	68.79 (9.3)	70.07 (10.3)
SES (%)		
- Low	16	15
- Below average	18	7
- Average	25	24
- Above average	27	28
- High	24	26
**Enabling factors patient**		
Supplementary insurance (%)	97	98
Mean travel time to hospital in minutes (SD)	27.30 (20.9)	27.82 (20.6)
Mean travel time to PT in minutes (SD)	2.48 (4.5)	2.60 (4.6)
Received non-TJA-related PT (%)	36	38
- mean number of non-TJA-related PT sessions between 6 to 12 months prior to surgery (SD)	10.92 (10.1)	10.53 (10.1)
**Enabling factors hospital** ***(n = 102)***		
Type of hospital %		
- General	41	42
- Specialized	4	3
- Academic	2	2
- Top clinical	41	45
- Independent treatment center	12	7
Mean procedure volume per hospital per year (SD)	855.62 (465.3)	873.13 (457.4)
**Enabling factors PT clinic** ***(n = 4347)***		
Mean number of all PT sessions claimed by PT clinics per year (SD)	6781.89 (6783.5)	6705.19 (6916.8)
Contract type PT %		
- Uncontracted	3	3
- Standard	3	3
- Basic	55	56
- Plus	38	37
**Need factors**		
Received TJA-related pre-operative PT %	46	51
- mean number of TJA-related PT sessions in 6 months prior to surgery (SD)	10.30 (10.6)	10.23 (10.4)
Received pre-operative homecare %	7	9
- mean number of hours of homecare received (SD)	9.27 (19.7)	6.53 (11.3)
Diabetes %	19	14
- Insulin %	5	3
- Other glucose-lowering drugs %	14	10
COPD %	17	15
Cardiovascular disease %	67	63
- Antihypertensives %	33	28
- Beta-blockers %	15	14
- Cholesterol-lowering agents %	6	6
- Anti-arrhythmia or vasoprotective agents %	13	15

Abbreviations: *SD* standard deviation, *PT* physical therapy, *TKA* total knee arthroplasty, *THA* total hip arthroplasty, *TJA* total joint arthroplasty, *SES* socioeconomic status, *COPD* chronic obstructive pulmonary disease

The large majority of patients (97% for TKA and 98% for THA) had a supplemental insurance (SI) plan (covering most or all of the out-of-pocket costs for PT sessions). Table 2 shows that patients with SI received significantly more PT sessions after surgery (63% more for TKA and 59% more for THA), were more often female, were older, received more non-TJA-related PT, more home care, more PT prior to surgery, and had more often cardiovascular disease, compared to patients without SI.

**Table 2:** Descriptive characteristics of patients who received TKA (*n* = 15,309) or THA (*n* = 14,325) by supplementary insurance status (2015–2018)

Characteristic	TKA patients		THA patients	
Supplemental insurance	With SI ***(n = 14,823)***	No SI ***(n = 486)***	With SI ***(n = 13,976)***	No SI ***(n = 349)***
Mean number of post-discharge PT sessions (SD)	20.96(11.2)	12.83(9.94)^**^	16.38(10.06)	10.59(9.59)^**^
**Predisposing factors**				
Female (%)	65	58^**^	66^*^	62^*^
Age (SD)	70.0(9.1)	66.5(9.4)^**^	70.9(9.8)	66.9(10.1)^**^
SES (%)				
- Low	16	13	15	18
- Below average	8	8	7	6
- Average	25	27	24	18
- Above average	27	25	28	27
- High	24	28	26	31
**Enabling factors**				
Mean travel time to hospital in minutes (SD)	27.31 (20.8)	27.30 (20.8)	27.40 (20.4)	27.12 (20.3)
Mean travel time to PT in minutes (SD)	2.48 (4.5)	2.47 (4.5)	2.61 (4.6)	2.57 (4.6)
Received non-TJA-related PT (%)	28	3^**^	38	6^**^
Type of hospital %				
- General	52	2	53	1
- Specialized	4	0	4	0
- Academic	1	0	1	0
- Top clinical	28	1	32	1
- Independent treatment center	12	0	8	0
Contract type PT %				
- Uncontracted	3	3	3	4
- Standard	3	3	3	4
- Basic	55	53	56	50
- Plus	38	40	37	42
**Need factors**				
Received TJA-related pre-operative PT %	47	9^**^	52	14^**^
Received pre-operative homecare %	31	23^**^	41	29^**^
Diabetes %	12	8	14	9
COPD %	17	11	21	17
Cardiovascular disease %	72	65^*^	68	58^**^

Abbreviations: *SD* standard deviation, *PT* physical therapy, *TKA* total knee arthroplasty, *THA* total hip arthroplasty, *TJA* total joint arthroplasty, *SES* socioeconomic status, *COPD* chronic obstructive pulmonary disease

^*^The distribution of the variable is significantly different between the groups of individuals with and without SI at *p* < 0.05

^**^The distribution of the variable is significantly different between the groups of individuals with and without SI at *p* < 0.001

### Regression analyses

The degree of primary care PT clinic clusters within hospitals was considered low. While 33% of PT clinics received patient referrals from only one hospital, 35% of PT’s received patient referrals from more than two hospitals. Additionally, only 19% of PT clinics provided post-discharge PT to more than 10 patients within the time frame of our study. Therefore, no corrections were performed for nesting of patients within PT clinics and for nesting of PT clinics within hospitals. Multilevel corrections were only performed for nesting of patients within hospitals.

All statistically significant (*p* < 0.05) predictors for post-discharge PT use after TJA are presented in Table 3 (TKA) and 4 (THA). In both groups the enabling factors were more strongly associated with post-discharge PT utilization than predisposing and need factors. The presence of SI increased the mean utilization of post-discharge PT the most (with 7.47 sessions for TKA patients and 5.72 sessions for THA patients). All other factors shown in Tables 3 and 4 were statistically significant in predicting the use of post-discharge PT as well, but the coefficients were smaller.

**Table 3:** Multilevel linear model predicting PT use after TKA surgery (2015–2018)

	Model A		Model B	
	β	SE	β	SE
**Predisposing factors**				
Sex (male)			0.38*	0.182
SES^a^	−0.28**	0.067	−0.28**	0.067
**Enabling factors**				
Supplementary insurance (yes)			7.47**	0.498
Contract type PT				
- Uncontracted (ref)			0	
- Standard			1.24	0.699
- Basic			1.62*	0.486
- Plus			1.63**	0.494
Number of non-TJA-related PT sessions between 6 to 12 months prior to surgery			0.11**	0.012
**Need factors**				
Number of TJA-related PT sessions in 6 months prior to surgery	0.20**	0.011	0.20**	0.011
Number of pre-operative homecare hours	−0.05*	0.016	− 0.05*	0.016
Constant	19.88**	0.318	11.02**	0.744
-2 log likelihood	116,840.3		116,240.1	

Abbreviations: *SE* standard error, *PT* physical therapy, *TKA* total knee arthroplasty, *TJA* total joint arthroplasty

*p < 0.05, **p < 0.001

^a^higher score means lower socioeconomic status

**Table 4:** Multilevel linear model predicting PT use after THA surgery (2015–2018)

	Model A		Model B	
	β	SE	β	SE
Enabling factors				
Supplementary insurance (yes)			5.72**	0.515
Contract type PT				
- Uncontracted (ref)			0	
- Standard			0.99	0.631
- Basic			1.87**	0.448
- Plus			2.21**	0.457
Number of non-TJA-related PT sessions between 6 to 12 months prior to surgery			0.15**	0.011
THA procedure volume			−0.002*	0.0005
**Need factors**				
Number of TJA-related PT sessions in 6 months prior to surgery	0.19**	0.010	0.18**	0.010
Diabetes: Insulin	1.07*	0.449		
COPD	0.60*	0.224	0.57*	0.223
Constant	14.74**	0.298	8.49**	0.786
-2 log likelihood	105,380.8		104,889.2	

Abbreviations: *SE* standard error, *PT* physical therapy, *THA* total hip arthroplasty, *TJA* total joint arthroplasty, *COPD* chronic obstructive pulmonary disease

*p < 0.05, **p < 0.001

### Evaluation of the model

The likelihood ratio test showed that the model improved significantly (*p* < 0.001) after including a random intercept at hospital level. The ICC was relatively low for both the TKA group (ICC = 0.04) and the THA group (ICC = 0.07) meaning that, respectively, 4 and 7% of variation in post-discharge PT utilization could be explained by differences on the hospital level.

### Sensitivity analyses

Despite the different coverage structure for post-discharge PT sessions in 2018, the percentage of patients with SI remained similar (96% in the TKA group and 98% in the THA group) (Table A1, Additional file 1). Statistically significant (*p* < 0.05) predictors for post-discharge PT use after TJA in 2018 are presented in Tables A2 (TKA) and A3 (THA) in the Additional file 1. Some differences in predicting factors and their effect on post-discharge PT utilization were found in 2018 compared to 2015–2018. In the TKA group the contract type of the PT clinic was no longer a statistically significant predictor and a slightly stronger effect was found for SES (β = − 0.41 to β = − 0.28). In the THA group the contract type of the PT clinic, the procedure volume and COPD were no longer statistically significant predictors, while insulin use in diabetes patients entered the model as a statistically significant need factor in the prediction of post-discharge PT utilization (β = 2.36). Although the large effect of SI slightly weakened in 2018 (β = 7.47 to β = 6.74 in TKA and β = 5.72 to β = 4.39 in THA), it remained the strongest predictor for PT utilization in both groups.

## Discussion

This study aimed to explore the assumption that historic claims data may not accurately reflect appropriate care, by investigating this in the context of post-discharge primary care PT use after TKA and THA for osteoarthritis patients, using multilevel linear regression analyses and then categorizing the predictors according to Andersen’s behavioral model [37]. In general, the results showed that post-discharge PT utilization was more significantly explained by enabling factors than by predisposing and need factors.

More specifically, the presence of SI for post-discharge PT care was the strongest predictor for PT use in both the TKA (β = 7.47) and THA (β = 5.72) group, which might suggest that the (insurance enabled) expressed demand for post-discharge PT care may play a bigger role for patients than their actual care needs [66]. This finding is convergent with previous studies’ findings of the association between having supplemental insurance and care utilization [46, 47, 67–72]. For example, Freburger and Holmes reported that patients with supplemental insurance were 31% more likely to receive PT, and received significantly more PT sessions, than patients without such insurance [73]. Similarly, Grana and Stuart reported that insurance status is a positive and statistically significant predictor of both initial access to care and the amount of (arthritis) care used [74]. No previous studies with clear divergent findings were found.

The results also show several other statistically significant predictors for the use of post-discharge PT, but their effects are smaller. Also noteworthy is that the results in Table 2 show that differences in SES between patients with or without SI are low, which could be interpreted as equal (financial) accessibility of the SI plan for patients in all SES groups.

### Limitations

First, it is important to note that TKA and THA are elective surgeries and the choice for a SI plan for post-discharge PT could have been contingent on expected use. In The Netherlands, each year all inhabitants have the possibility to opt in or out of a SI plan that covers PT care (i.e. the first 20 post-discharge PT sessions after TKA or THA are not covered by the government regulated basic health insurance plan). For these SI plans there is no waiting time and no medical selection. As a consequence, part of the effect of having a SI plan on the utilization of post-discharge PT could potentially be explained by patients who have selected insurance coverage based on their anticipated behavioral (“selection on moral hazard”) response to insurance [75]. For example Holst et al. found that for 54% of Dutch study participants (*n* = 885), the fact that they expected to need care in the following year that was included in a SI plan, played a (very) important role in buying that SI plan [68]. The degree to which this phenomenon occurred in this study is unknown and therefore the impact on the results is unclear. However, because of the possibility of self-selection, we have corrected for differences in patient characteristics between those with and those without supplemental insurance using a broad range of characteristics in our statistical models. In the models adjusted for these characteristics, the coefficients of the variable ‘having supplemental insurance’ are still relatively large and strongly significant.

Second, although this study categorized 16 different potential predictors of PT utilization in the 3 components (i.e. predisposing, enabling and need factors) of Andersen’s behavioral model, there are some other relevant potential predictors that could have been included as well. For example: health beliefs (e.g. attitudes, values and knowledge related to post-discharge PT services), having overweight or obesity, and the specific type of PT session (e.g. individual or group session). Including these factors could have improved the accuracy of the models, but were unavailable in the data.

Third, in the TKA and THA groups 97 and 98% of patients, respectively, had a SI plan. This is higher than the 84.2% (2015) to 83.7% (2018) of people with SI in the total (insured) Dutch population [76, 77]. One explanation for this might be the higher-than-average age of our study sample who, therefore, are more likely to have a SI plan. Another explanation might be found in the 9% of patients in the TKA group and 17% of patients in the THA group who were excluded from the study sample because they did not receive any post-discharge PT care based on the insurer’s claims data. It is possible that these patients chose not to use post-discharge PT care for financial reasons (e.g. because they did not have a SI plan), or that there was no need or want for PT care, or that they received post-discharge PT in another (long-term) care setting. Likewise, due to incomplete data on primary care PT, we could not fully observe the use of PT for a small group of patients, for example because they switched to another health insurance company. These patients might have behaved differently and could have had an effect on our estimations. The direction and magnitude of this potential bias is unclear.

Fourth, having a SI plan for post-discharge PT was characterized as a dichotomous (yes/no) variable. However, during the years of our study period, there were actually four (2015–2017) or three (2018) different SI plans with different coverage for TJA-related post-discharge PT. From 2015 to 2017 the four SI plans offered coverage for 9, 12, 27 or 40 PT sessions per year, respectively. In 2018 the insurer’s reimbursement policy changed and three different SI plans instead of four were offered. Although these three plans had clear mutual differences in their coverage for PT (and other types of care), they all covered 20 PT sessions per year for TJA-related post-discharge PT [62–65]. Since, from the 21st post-discharge PT session onwards, all additional TJA-related post-discharge PT sessions were reimbursed by the Dutch (mandatory) basic health insurance [78], the patients with a 9 or 12 session coverage SI plan might still have experienced some degree of financial incentive in restraining their PT use. The data of this current study lacked detailed information on this and future studies should aim to include these types of complicated variations in coverage in their analyses to improve the accuracy of results.

Fifth, this study provides no information on the quality outcomes of PT treatments. More intense post-discharge PT may prevent complications and result in a higher functional status and lower degree of residual complaints in some patients [22, 79, 80]. Some studies have even suggested that PT is especially effective in the later stages of rehabilitation. This is because in the first few months after surgery, the joint is still healing, and exercises cannot be performed with sufficient intensity to reduce limitations in mobility and physical functioning [81]. To determine the appropriate average number of post-discharge PT sessions after TJA, future studies and bundled payment pricing methods should also take the quality aspect into account [6].

### Implications of the results

Although bundled payment contracts can incentivize providers to reduce unwarranted variations in lower extremity joint replacement spending [4, 5], this study shows that if enabling factors (such as the supplemental insurance coverage or co-payments) are not taken into account in risk-adjustment of the bundle price, they may cause (currently frequently used) historic claims-based pricing methods to over- or underestimate appropriate post-discharge PT use.

When determining an appropriate price for the bundle, estimating a prediction model can provide valuable insights into the desirable and undesirable effects of using casemix factors. While (supplemental) insurance coverage might result in overuse due to (patient-induced or supplier-induced) moral hazard, a deductible or co-payment might result in underuse. Not taking into account these factors may result in bundle prices that are either too high or too low. If bundle prices are too high, the margins of providers will be high, and payers and patients will not benefit from the changes as intended. Conversely, if bundle prices are too low, providers are less likely to participate in the bundled payment contract, or they may drop out when they can, which would also limit the potential benefits for payers and patients [8]. Therefore, while using historical claims data to determine the price for a bundle of care is crucial to give payers and providers some understanding of the resources necessary to deliver high-quality care and optimal outcomes, not adjusting that bundle price for all relevant casemix factors is a risk because it can hamper the successful implementation of the bundled payment contract and the desired changes in care delivery it aims to support. Given that relevant casemix factors (like supplemental insurance status in the case of Dutch primary PT care) are usually not included in standard risk-adjustment models, it would take an extra effort to apply.

Given the limitations of historic claims data, using it as a single source to set the price of a bundle is a risk that could potentially be reduced by considering other (complementary) pricing approaches. One way would be to better-leverage clinical guidelines and best practice standards. For example, the science-based PT guideline for hip and knee arthrosis of the Dutch Association for Physical Therapy states that post-discharge PT care should be limited to only teaching patients some exercises that they can perform independently at home. Longer PT treatments (i.e. ‘starting off with 1 to 2 sessions per week for 8 to 12 weeks and then decreasing the number of sessions per week during the course of the treatment period’) ‘should only be considered if there is an increased risk of delayed recovery and / or complications’ [82]. The guideline then describes 12 of these risk factors (e.g. overweight, high pain score or psychosocial functioning), but unfortunately does not provide information about how often these risks are present in TKA and THA patients. Nevertheless this might be relatively easy to determine in existing data. Then, the (scientific guideline-based) average number of post-discharge PT sessions could be calculated and leveraged as an additional source of information to determine the appropriate price for the bundle (i.e. a source that is unbiased by potential supplier induced demand and moral hazard effects).

The bundle price could also be based on the average number of post-discharge PT sessions for TKA and THA patients in the more efficient (best practice) providers instead of on the average number of sessions of all providers. Also, the performance data of those higher performing providers could be made publicly available so that all providers can see, for example, that achieving the quality outcomes needed to earn savings under the set bundle price is practically feasible. The bundle price could then be revised over time to ensure continual improvement by both the higher and lower performing providers, and to continue decreasing unwarranted variations in their care delivery.

Finally, the issue of using historical claims data which inaccurately reflect appropriate care is much broader than with respect to post-discharge PT. For example, as recent estimates have shown, the cost of waste in the US health care system ranges from $760 billion to $935 billion, which is approximately 25% of its total health care spending [83]. Much of this waste (like overtreatment and low-value care) will be reflected in historic claims data. Since many provider payment systems will transition towards Alternative Payment Models in the coming years, and new prices have to be determined for populations and bundles of care, we believe that there is an opportunity there to explore alternative pricing approaches that better align with value-based care delivery.

## Conclusion

This study shows that if enabling factors (such as supplemental insurance coverage or co-payments) are not taken into account in risk-adjustment of the bundle price, they may cause historic claims-based pricing methods to over- or underestimate appropriate post-discharge PT use, which would result in a bundle price that is either too high or too low. In order to prevent this, estimating a prediction model that includes a broad set of potentially relevant casemix variables can provide valuable insights into their desirable and undesirable effects on the utilization of care.

## Supplementary Information

**Additional file 1: Table A1.** Descriptive characteristics of patients who received physical therapy after knee or hip replacement surgery (2018). **Table A2.** Multilevel linear model predicting PT use after TKA surgery (2018). **Table A3.** Multilevel linear model predicting PT use after THA surgery (2018).

## Data Availability

The data that support the findings of this study are available from Dutch insurance company Zilveren Kruis but restrictions apply to the availability of these data, which were used under license for the current study, and so are not publicly available. Data are however available from the corresponding author (s.steenhuis@vu.nl) upon reasonable request and with permission of Zilveren Kruis.

## Abbreviations

PT: Physical therapy
TKA: Total knee arthroplasty
THA: Total hip arthroplasty
TJA: Total joint arthroplasty
DRG: Diagnosis related group
CBS: Statistics Netherlands (Centraal Bureau voor de Statistiek)
COPD: Chronic obstructive pulmonary disease
SI: Supplemental insurance
PCG: Pharmaceutical cost group
VIF: Variance inflation factor
ICC: Intraclass correlation coefficient
SD: Standard deviation
SE: Standard error
SES: Socioeconomic status
