# Supplementary material for: The potential risk of using historic claims to set bundled payment prices: the case of physical therapy after lower extremity joint replacement
Source: BMC Health Serv Res. 2022 Aug 19;22:1061. doi: 10.1186/s12913-022-08410-7 (PMC9392222; doi:10.1186/s12913-022-08410-7)
Supplement: Supplementary file 1 — Additional file 1: Table A1. Descriptive characteristics of patients who received physical therapy after knee or hip replacement surgery (2018). Table A2. Multilevel linear model predicting PT use after TKA surgery (2018). Table A3. Multilevel linear model predicting PT use after THA surgery (2018). [file 12913_2022_8410_MOESM1_ESM.docx]

**Additional file 1**

| **Table A1: Descriptive characteristics of patients who received physical therapy after knee or hip replacement surgery (2018)** | | |
| --- | --- | --- |
| **Characteristic** | **TKA *(n=4,226)*** | **THA *(n=4,013)*** |
| Mean number of post-discharge PT sessions (SD) | 20.37 (11.11) | 16.42 (10.05) |
| **Predisposing factors** |  |  |
| SES % |  |  |
| - Low | 13 | 13 |
| - Below average | 9 | 8 |
| - Average | 26 | 24 |
| - Above average | 27 | 28 |
| - High | 25 | 27 |
| **Enabling factors** |  |  |
| Supplementary insurance % | 96 | 98 |
| **Need factors** |  |  |
| Received pre-operative PT % | 45 | 51 |
| Received pre-operative homecare % | 7 | 8 |
| Received non-TJA related PT % | 39 | 42 |
| Diabetes % | 18 | 13 |
| - Insulin % | 5 | 3 |
| - Other glucose-lowering drugs % | 14 | 10 |
| COPD % | 16 | 15 |
| Cardiovascular disease % | 66 | 62 |

Abbreviations: SD, standard deviation; PT, physical therapy; TKA, total knee arthroplasty; THA, total hip arthroplasty; TJA, total joint arthroplasty; SES, socioeconomic status; COPD, chronic obstructive pulmonary disease.

| **Table A2: Multilevel linear model predicting PT use after TKA surgery (2018)** | | |
| --- | --- | --- |
|  | **β** | **SE** |
| **Predisposing factors** |  |  |
| SES ^†^ | -0.41* | 0.129 |
| **Enabling factors** |  |  |
| Supplementary insurance (yes) | 6.74** | 0.875 |
| **Need factors** |  |  |
| Number of pre-operative homecare hours | -0.07* | 0.033 |
| Number of pre-operative PT sessions | 0.21** | 0.026 |
| Number of non-TJA PT sessions in prior year | 0.19** | 0.027 |
| Constant | 13.36** | 0.945 |
| -2 log likelihood | 32,012.8 | |

*p<0.05, **p<0.001

^†^ higher score means lower socioeconomic status

Abbreviations: SE, standard error; PT, physical therapy; TKA, total knee arthroplasty; TJA, total joint arthroplasty; SES, socioeconomic status.

| **Table A3: Multilevel linear model predicting PT use after THA surgery (2018)** | | |
| --- | --- | --- |
|  | **β** | **SE** |
| **Enabling factors** |  |  |
| Supplementary insurance (yes) | 4.39** | 0.982 |
| **Need factors** |  |  |
| Diabetes: Insulin | 2.36* | 0.868 |
| Number of pre-operative PT sessions | 0.18** | 0.023 |
| Number of non-TJA PT sessions in prior year | 0.23** | 0.024 |
| Constant | 10.15** | 0.997 |
| -2 log likelihood | 29,008.9 |  |

*p<0.05, **p<0.001

Abbreviations: SE, standard error; PT, physical therapy; THA, total hip arthroplasty; TJA, total joint arthroplasty.
